# Genomic profiling of rectal adenoma and carcinoma by array-based comparative genomic hybridization

**DOI:** 10.1186/1755-8794-5-52

**Published:** 2012-11-16

**Authors:** Zhi-Zhou Shi, Yue-Ming Zhang, Li Shang, Jia-Jie Hao, Tong-Tong Zhang, Bo-Shi Wang, Jian-Wei Liang, Xi Chen, Ying Zhang, Gui-Qi Wang, Ming-Rong Wang, Yu Zhang

**Affiliations:** 1 State Key Laboratory of Molecular Oncology, Cancer Institute /Hospital, Peking Union Medical College and Chinese Academy of Medical Sciences, Beijing, China; 2 Department of Endoscopy, Cancer Institute (Hospital), Peking Union Medical College and Chinese Academy of Medical Sciences, Beijing, China; 3 Department of Abdominal Surgery, Cancer Institute/Hospital, Peking Union Medical College and Chinese Academy of Medical Sciences, Beijing, China

## Abstract

**Background:**

Rectal cancer is one of the most common cancers in the world. Early detection and early therapy are important for the control of death caused by rectal cancer. The present study aims to investigate the genomic alterations in rectal adenoma and carcinoma.

**Methods:**

We detected the genomic changes of 8 rectal adenomas and 8 carcinomas using array CGH. Then 14 genes were selected for analyzing the expression between rectal tumor and paracancerous normal tissues as well as from adenoma to carcinoma by real-time PCR. The expression of GPNMB and DIS3 were further investigated in rectal adenoma and carcinoma tissues by immunohistochemistry.

**Results:**

We indentified ten gains and 22 losses in rectal adenoma, and found 25 gains and 14 losses in carcinoma. Gains of 7p21.3-p15.3, 7q22.3-q32.1, 13q13.1-q14.11, 13q21.1-q32.1, 13q32.2-q34, 20p11.21 and 20q11.23-q12 and losses of 17p13.1-p11.2, 18p11.32-p11.21 and 18q11.1-q11.2 were shared by both rectal adenoma and carcinoma. Gains of 1q, 6p21.33-p21.31 and losses of 10p14-p11.21, 14q12-q21.1, 14q22.1-q24.3, 14q31.3-q32.1, 14q32.2-q32.32, 15q15.1-q21.1, 15q22.31 and 15q25.1-q25.2 were only detected in carcinoma but not in adenoma. Copy number and mRNA expression of EFNA1 increased from rectal adenoma to carcinoma. C13orf27 and PMEPA1 with increased copy number in both adenoma and carcinoma were over expressed in rectal cancer tissues. Protein and mRNA expression of GPNMB was significantly higher in cancer tissues than rectal adenoma tissues.

**Conclusion:**

Our data may help to identify the driving genes involved in the adenoma-carcinoma progression.

## Background

Rectal cancer is the 5th leading cause of cancer-related death and its incidence is increasing at a rate of 4.2% per year in China [1]. Early detection and early therapy are important for the control of death caused by rectal cancer.

The majority of epithelial cancers arise through a stepwise progression from normal cells, through dysplasia, into malignant cells that have invasive and metastatic potential. The classic example of this process is the colorectal adenoma to carcinoma progression [2,3]. Genomic aberrations are found frequently in cancers and are believed to contribute to initiation and progression of cancer by deletion-induced down-expression of tumor suppressor genes or amplification and activation of oncogenes. In colorectal cancer the most frequent chromosomal aberrations were gains at 7p, 7q, 8q, 13q, and 20q and losses of 1p, 4p, 4q, 5q, 8p, 14q, 15q, 17p and 18q [4-9]. In particular, 8q, 13q and 20q gains and 8p, 15q and 18q losses are linked with colorectal adenoma to carcinoma progression. However, most of published reports are focused on colon cancer. Little information is available concerning the genomic aberrations of rectal carcinoma, especially DNA copy number changes in the progression from adenoma to tumor.

In the present study, we investigated the genomic aberrations of rectal adenoma and carcinoma by oligonucleotide-based array CGH, and identified common and different alterated chromosome regions between rectal adenoma and carcinoma. Then the expression of 15 genes at selected chromosome regions above was analyzed by real-time PCR or immunohistochemistry.

## Methods

### Patients and samples

Biopsy tissues from 22 rectal adenoma patients and 36 rectal carcinoma patients were collected by the Department of Endoscopy, Cancer Hospital, Peking Union Medical College and Chinese Academy of Medical Sciences, Beijing, China. Biopsy samples were obtained by colonoscopy and stored at −80°C. Definitive pathological result from a biopsy was obtained at a later clinical course. An experienced pathologist confirmed that normal cell content of all the samples was less than 40% by HE staining. All the samples used in this study were residual specimens after diagnosis sampling. And all patients signed separate informed consent forms for sampling and research. The clinicopathological characteristics of the patients in array CGH assay are summarized in Table 1.

**Table 1:** Clinical Characteristics of 16 Patients Studied by Array CGH

**Case No.**	**Sex**	**Age**	**Type**	**Location**
1	F	52	Adenoma	Rectum
2	F	49	Adenoma	Rectum
3	M	75	Adenoma	Rectum
4	M	47	Adenoma	Rectum
5	M	57	Adenoma	Rectum
6	F	61	Adenoma	Rectum
7	M	69	Adenoma	Rectum
8	F	75	Adenoma	Rectum
9	M	69	Carcinoma	Rectum
10	M	61	Carcinoma	Rectum
11	F	70	Carcinoma	Rectum
12	F	73	Carcinoma	Rectum
13	M	42	Carcinoma	Rectum
14	M	32	Carcinoma	Rectum
15	F	31	Carcinoma	Rectum
16	M	66	Carcinoma	Rectum

### Genomic DNA extraction and array-based CGH

Genomic DNA was isolated from tumor tissues using the Qiagen DNeasy Blood & Tissue Kit as described by the manufacturer (Qiagen, Hilden, Germany).

Array CGH experiments were performed using standard Agilent protocols (Agilent Technologies, Santa Clara, CA). Commercial human genomic DNA (PROMEGA, Warrington, UK) was used as reference. For each CGH hybridization, 500 ng of reference genomic DNA and the same amount of tumor DNA were digested with Alu I and RSA I restriction enzyme (PROMEGA, Warrington, UK). The digested reference DNA fragments were labeled with cyanine-3 dUTP and the tumor DNA with cyanine-5 dUTP (Agilent Technologies, Santa Clara, CA). After clean-up, reference and tumor DNA probes were mixed and hybridized onto Agilent 44K human genome CGH microarray (Agilent) for 40 h. Washing, scanning and data extraction procedures were performed following standard protocols.

Array CGH data set is available at Gene Expression Omnibus (GEO) http://www.ncbi.nlm.nih.gov/geo/[10], accession number GSE34472.

### Microarray data analysis

Microarray data were analyzed using Agilent Genomic Workbench (Agilent Technologies, Santa Clara, CA) and MD-SeeGH (http://www.flintbox.ca). The Aberration Detection Method 2 algorithm with threshold at 6 (Agilent Genomic Workbench) was applied to identify common genomic aberrations. Mean Log2^ratio^ of all probes in a chromosome region between 0.125 and 0.5 was classified as genomic gain, > 0.5 as high-level DNA amplification, < −0.125 as hemizygous loss, and < −0.5 as homozygous deletion. Minimal regions of gains or losses in our study defined as the smallest overlapping aberrant chromosomal regions identified by Agilent Genomic Workbench. Frequency plot comparison method (MD-SeeGH) was used to compare frequency of DNA copy number changes between rectal adenoma and carcinoma.

### Total RNA extraction and real-time PCR

Total RNA was isolated from tissues using the RNeasy Mini Kit as described by the manufacturer (Qiagen, Hilden, Germany).

The PCR reactions were performed in a total volume of 20 μl, including 10 μl of 2 X SYBR ® Green PCR Master Mix (Applied Biosystems, Warrington, UK), 2 μl of cDNA (5 ng/μl), 1 μl of primer mix (10 μM each). The PCR amplification and detection were carried out in a 7300 Real Time PCR System (Applied Biosystems) for 45 cycles, each with 15 s at 95 °C, 1 min at 60 °C, and initial denaturation with 10 min at 95 °C. The relative gene expression was calculated using the comparative CT Method [11]. The copy number of the target gene normalized to an endogenous reference (GAPDH), and relative to calibrator was given by the formula 2 − ΔΔCt. ΔCT was calculated by subtracting the average GAPDH CT from the average CT of the gene of interest. The ratio defines the level of relative expression of the target gene to that of GAPDH.

### Immunohistochemical staining

Formalin-fixed, paraffin-embedded specimens of rectal adenoma and carcinoma were detected in immunohistochemistry assay. Tissues of each case were repeated for three times. The slides were deparaffinized, rehydrated, immersed in 3% hydrogen peroxide solution for 10 min, heated in citrate buffer (pH 6) for 25 min at 95°C, and cooled for 60 min at room temperature. The slides were blocked by 10% normal goat serum for 30 min at 37°C and then incubated with rabbit polyclonal antibody against DIS3 (PTGLab), rabbit polyclonal antibody against GPNMB (PTGLab) overnight at 4°C. After being washed with PBS, the slides were incubated with biotinylated secondary antibody (diluted 1:100) for 30 min at 37°C, followed by streptavidin-peroxidase (1:100 dilution) incubation for 30 min at 37°C. Immunolabeling was visualized with a mixture of 3,3'-diaminobenzidine solution. Counterstaining was carried out with hematoxylin.

Expression level was determined on the basis of staining intensity and percentage of immunoreactive cells. Negative expression (score = 0) was no or faint staining, or moderate to strong staining in <25% of cells. Weak expression (score = 1) was a moderate or strong staining in 25% to 50% of cells. And strong expression (score = 2) was > 50% of the cells with strong staining. Weak expression and strong expression defined as positive staining.

### Statistical analysis

Statistical analyses were conducted using the Student’s t-test and performed with the statistical software SPSS 15.0. The differences were judged as statistically significant when the corresponding two-sided P value were <.05.

## Results

### Recurrent copy number alterations in rectal adenoma and carcinoma detected by array CGH

**Figure 1:**
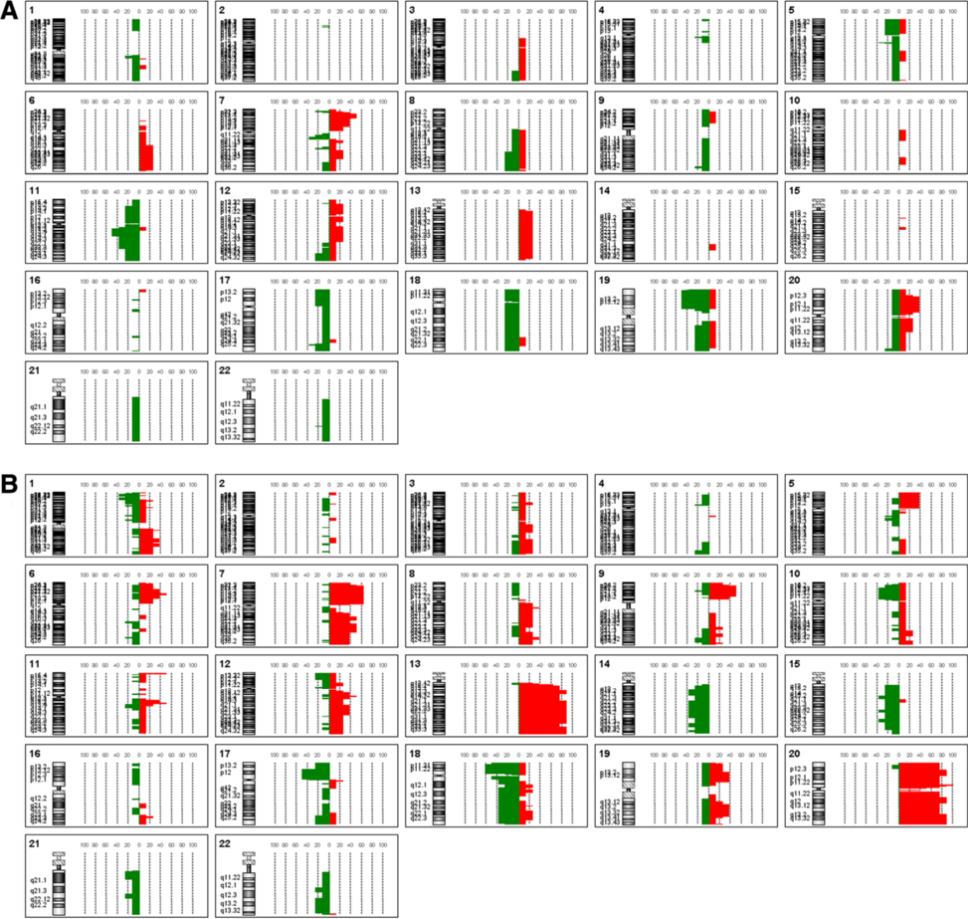
**Genome-wide frequency plot of rectal adenoma (A) and adenocarcinoma (B) in array CGH assay.** Line on the right of 0%-axis: gain; Line on the left of 0%-axis: loss.

Seven out of eight adenomas and all of carcinomas had genomic aberrations. More alterations were observed in patients of rectal cancer than adenoma, and the numbers of changes were 39.13±20.48 and 14.3±6.164, respectively (Additional file 1: Figure S1). Array CGH results showed that the most frequent copy number alterations in rectal adenoma were gains of 7p21.3-p15.3 and 20p12.3-p11.21 and losses of 5q13.2, 7q11.23, 11q13.1-q14.1, 17q25.1 and 19p13.3-p13.11 (Figure 1A, Tables 2 and 3). And the most common genetic aberrations in rectal carcinoma were gains of 7p21.3-p15.3, 7p15.3-p14.1, 7p14.1-p13, 7p13-p11.2, 13q13.1-q14.11, 13q21.1-q32.1, 13q32.1-q34, 20p11.21, 20q11.23-q12 and 20q13.2-q13.33 and losses of 17p13.1-p11.2, 18p11.32-p11.21 and 18q11.1-q11.2 (Figure 1B, Tables 2 and 3).

**Table 2:** Genomic Gains in Rectal Adenoma and Adenocarcinoma

**Chromosome Region**	**Rectal adenoma**				**Rectal adenocarcinoma**			
	**Start**	**End**	**No. of probes**	**No. of cases**	**Start**	**End**	**No. of probes**	**No. of cases**
1q21.3					150819451	150852905	3	3
1q25.3-q31.3					183720174	197184608	157	3
1q32.1-q41					204180950	214439909	173	3
5p13.3-p12					33503866	45681293	165	3
6p21.33-p21.31					30737615	33655570	151	4
6q16.3-q27	100547312	168205989	848	2				
7p21.3-p15.3	11041844	23202043	119	4	7671318	23172047	142	5
7p15.3-p14.1					23821348	39813908	231	5
7p14.1-p13					40099046	44497196	64	5
7p13-p11.2					44890654	55242365	111	5
7q21.11-q21.12	81196827	86205180	42	2				
7q21.12-q21.3					87207024	97321855	144	4
7q22.3-q32.1	106191096	127234809	245	2	105253205	127519635	260	4
8q12.1					59565778	61340797	21	3
8q24.21-q24.22					128816904	133653633	42	3
9p24.1-p21.1					7058096	31463899	251	4
11p15.5					192958	2278596	76	4
11q13.2					66917525	67689856	30	4
12p13.31-p11.21	9053548	30700931	337	2				
12q12-q13.11	37052371	47174877	139	2				
12q13.13					50568352	51486634	34	4
12q14.1-q22	57350276	91428773	354	2				
13q13.1-q14.11 13q21.1-q32.1 13q32.3-q34	21038984	109780488	909	2	32490193	39679219	79	7
					52774228	94079000	275	7
					100091512	114022929	148	7
19p13.2-p13.11					9800520	19631574	473	3
19q13.13-q13.33					43396893	55615310	550	3
20p11.21	7296794	23132344	189	3	22510206	23380542	15	8
20q11.23-q12	29592072	42681834	275	2	35467169	41087006	78	7
20q13.2-q13.33					52017030	62323759	215	7

Note: The number of rectal adenoma and adenocarcinoma in Array CGH study are both 8 cases.

**Table 3:** Genomic Losses in Rectal Adenoma and Adenocarcinoma

**Chromosome Region**	**Rectal adenoma**				**Rectal adenocarcinoma**			
	**Start**	**End**	**No. of probes**	**No. of cases**	**Start**	**End**	**No. of probes**	**No. of cases**
1p36.23-p36.22					7804415	11633739	82	3
1p36.22-p36.13					12600054	16167534	41	3
1p36.12-p35.3					21802142	29525663	226	3
1q21.2-q21.3	148163183	149505863	60	2				
1q21.3-q23.1	151880217	155031244	154	2				
4q12	55913547	57653302	38	2				
5p15.33-p12	260981	45865412	433	2				
5q13.2	68434643	68900029	18	3				
7p22.2-p22.1	4298590	6547570	42	2				
7q11.23	72003839	75977276	77	3				
7q22.1	99538250	101895994	79	2				
8q22.2-q24.3	100781187	143914353	448	2				
8q24.3	143914353	146250824	75	2				
9q34.11	130111425	132321365	64	2				
10p14-p11.21					11825924	35645512	315	3
11p15.2-p11.12	14750051	50638829	468	2				
11q13.1-q14.1	63802950	80046693	442	4				
12q24.23-q24.33	116956235	132193660	257	2				
14q12-q21.1					30209271	38927323	130	3
14q22.1-q24.3					48874529	77750644	544	3
14q31.3-q32.1					87763614	93260389	110	3
14q32.2-q32.32					99254905	102592287	70	3
15q15.1-q21.1					38653893	42843706	119	3
15q22.31					61519869	64628895	74	3
15q25.1-q25.2					76206143	79967204	77	3
17p13.1-p11.2	84287	21386319	606	2	8327645	20974722	266	4
17q25.1	70528777	71603516	61	3				
18p11.32-p11.21	170229	13875315	173	2	2580000	13752309	137	5
18q11.1-q11.2	16904187	76018409	684	2	16976046	20313378	51	4
19p13.3-p13.11	1432408	19699544	795	4				
19q13.11-q13.43	37554715	63672832	1114	2				
20q13.33	60039825	62320720	85	2				
22q13.1	37689058	37715431	3	2				

Note: The number of rectal adenoma and adenocarcinoma in Array CGH study are both 8 cases.

### Common and distinct genomic events in rectal adenoma and carcinoma

**Figure 2:**
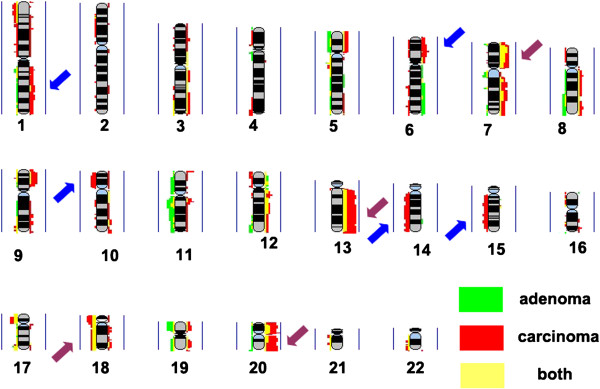
**Frequency plot comparison of rectal adenoma and carcinoma.** Red: carcinoma; green: adenoma; yellow: shared by both. The presentation is per array probe; gains and losses are represented by the colors on the right and left, respectively. Vertical blue line represents 100% of the samples. Brown and blue arrows highlight the changed chromosomal areas that were common or distinct between rectal adenoma and carcinoma, respectively.

By comparing the genomic aberrations of rectal adenoma and carcinoma, we found that gains of 7p21.3-p15.3, 7q22.3-q32.1, 13q13.1-q14.11, 13q21.1-q32.1, 13q32.3-q34, 20p11.21 and 20q11.23-q12 and losses of 17p13.1-p11.2, 18p11.32-p11.21, and 18q11.1-q11.2 were shared by rectal adenoma and carcinoma. However, gains of 1q, 6p21.33-p21.31 and losses of 10p14-p11.21, 14q12-q21.1, 14q22.1-q24.3, 14q31.3-q32.1, 14q32.2-q32.32, 15q15.1-q21.1, 15q22.31 and 15q25.1-q25.2 were detected in carcinoma but not in adenoma (Figure 2, Tables 2 and 3).

### Candidate target genes of interesting gains and losses

**Figure 3:**
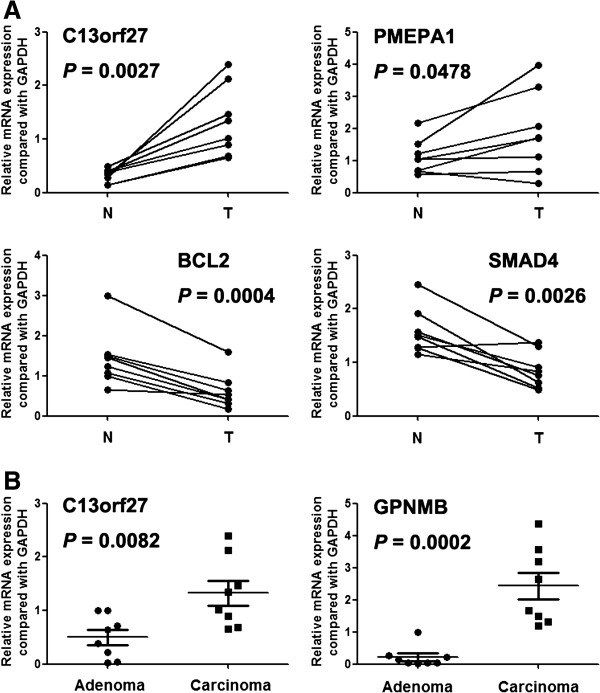
**Expression of genes which were located on the common aberrant chromosomal regions in rectal adenoma and carcinoma**. N: paracancerous normal tissues; T: rectal cancer tissues.

Further, we selected 14 genes of 1q, 6p, 7p, 13q, 18q and 20q to analyze the mRNA expression by real-time PCR (Table 4). Array CGH found that copy number increase of GPNMB (7p15.2), OXGR1 (13q32.1), C13orf27 (13q32.2-q34), PMEPA1 (20q13.31), PHACTR3 (20q13.32) and decrease of SMAD4 (18q21.2), BCL2 (18q21.33) occurred in both rectal adenoma and carcinoma. Our real-time PCR results showed that C13orf27 and PMEPA1 were overexpressed in rectal cancer tissues comparing with paracancerous normal tissues. BCL2 and SMAD4 were underexpressed in tumor tissue (Figure 3A). And the expression level of C13orf27 and GPNMB was significantly higher in cancer tissues than rectal adenoma tissues (Figure 3B).

**Table 4:** Primers of genes in Real-time PCR assay

**Gene**	**Forward primer**	**Backward primer**	**Size (bp)**
GAPDH	GGTCGTATTGGGCGCCTGGTC	TGACGGTGCCATGGAATTTGCCA	148
KIFC1	TCTCTGGGTGGTAGTGCTAAGA	TAAGTCACTTCCTGTTGGCCTG	148
SOX4	GACCGGGACCTGGATTTTAACT	TGAAAACCAGGTTGGAGATGCT	133
PBX2	AAGTTCCAAGAGGAGGCAAACA	TCCTGAGAGATTGAAAGAGCCG	132
ESRRG	GCTATCCTGCAGCTGGTAAAGA	GCTATCCTGCAGCTGGTAAAGA	133
KDM5B	CCCTCAGACACATCCTATTCCG	CAGTCCACCTCATCTCCTTCTG	101
PTGS2	TGTATCCTGCCCTTCTGGTAGA	AAGGAGAATGGTGCTCCAACTT	85
EFNA1	GTGGCAAAATCACTCACAGTCC	CTATGTAGAACCCGCACCTCTG	91
BCL2	AGGATTGTGGCCTTCTTTGAGT	CGGTTCAGGTACTCAGTCATCC	113
SMAD4	TGTTGATGACCTTCGTCGCTTA	ATGCTCTGTCTTGGGTAATCCG	81
PHACTR3	TATGACAGGAGGGCAGACAAAC	GCTTGCTTGATGCATGTACCTC	118
C13orf27	TCAGGCTCAGCAGATGAAATGT	TCCAGTGGATTTTATGGGGAGC	85
PMEPA	CTGAGCCACTACAAGCTGTCTG	CTTCTGAGGACAGGGCATCTTC	85
OXGR1	ATCTTGAGGGTCATTCGGATCG	TGTCGCTGACCACCACATATAG	148
GPNMB	GTCACTGTGATCTCCCTCTTGG	TTTGCACGGTTGAGAAAGACAC	116

**Figure 4:**
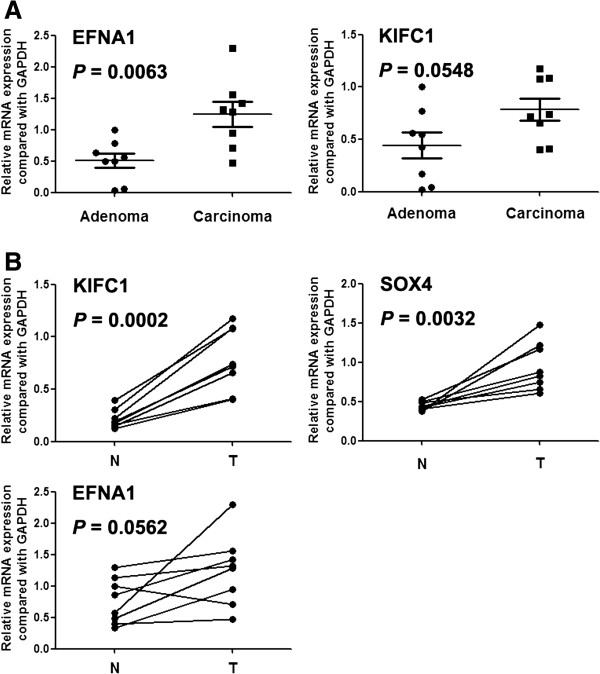
**Expression of genes which were located on the distinct aberrant chromosomal regions in rectal adenoma and carcinoma.** N: paracancerous normal tissues; T: rectal cancer tissues.

Copy number increase of EFNA1 (1q22), PTGS2 (1q31.1), KDM5B (1q32.1), ESRRG (1q41), KIFC1 (6p21.32), PBX2 (6p21.32) and SOX4 (6p22.3) were only detected in rectal cancer in array CGH. Among them, EFNA1 had increased expression in carcinoma compared with adenoma, and KIFC1 had an upward trend but not significant in statistical analysis (Figure 4A). Of these genes KIFC1 and SOX4 were also significantly overexpressed in rectal tumor tissues than paracancerous tissues (Figure 4B).

**Figure 5:**
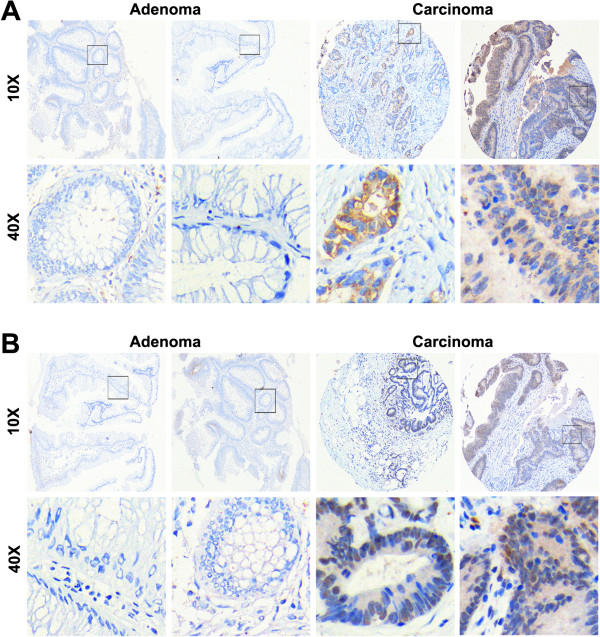
Expression of GPNMB and DIS3 by immunohistochemistry assay.

We also analyzed the protein expression of GPNMB (7p15.2) and DIS3 (13q22.1) by immunohistochemistry. Of all six detected rectal adenoma tissues, GPNMB and DIS3 had no expression. In twenty rectal cancer tissues, GPNMB and DIS3 were positively stained in six and five cases, respectively (Figure 5).

## Discussion

In the past decades, a number of genomic changes were found in colorectal adenoma and carcinoma, but the target genes are limited and molecular mechanism of adenoma to carcinoma progression is still unknown.

Previous studies found that 8q, 13q and 20q gains and 8p, 15q and 18q losses are linked with colorectal adenoma to carcinoma progression [4-9]. Our study narrowed down the gain regions to 13q13.1-q14.11, 13q21.1-q32.1, 13q32.2-q34 and 20q11.23-q12 and the loss regions to 18q11.2. Furthermore, gains of 7p21.3-p15.3 and 7q22.3-q32.1 and losses of 17p13.1-p11.2, 18p11.32-p11.21 were also found in both rectal adenoma and carcinoma.

Our study also showed that some genomic aberrations were present in rectal tumor but not in adenoma. They are gains of 1q and 6p21.33 and losses of 10p14-p11.21, 14q12-q21.1, 14q22.1-q24.3, 14q31.3-q32.1, 14q32.2-q32.32, 15q15.1-q21.1, 15q22.31 and 15q25.1-q25.2. These aberrations occurred at the later stages of rectal carcinogenesis, and may contribute the progression from adenoma to carcinoma.

Identifying the candidate targets underlying the genomic aberrations was important for understanding the mechanism of carcinogenesis. Carvalho et al. found that the overexpressions of C20orf24, AURKA, RNPC1, TH1L, ADRM1, C20orf20 and TCRL5 in carcinomas compared with adenomas were correlated with 20q gain [4]. Habermann et al. showed that copy number changes of 7q, 8p, 8q, 13q, 18p, 18q, 20p and 20q deregulated the average expression levels of the genes on these chromosome arms [12]. However, most of samples detected in these reports were colon cancer which had some different genomic aberrations compared with rectal cancer [13], expression-dysregulated genes in the carcinogenesis of rectum were still limited. By literature analyses, we selected 14 genes to compare their expression between in tumor and paracancerous tissues or between in rectal adenoma and carcinoma tissues. Of them, copy number and mRNA expression of EFNA1 increased from rectal adenoma to carcinoma, and C13orf27 and PMEPA1 with gains in both adenoma and carcinoma were overexpressed in rectal cancer tissues. These results revealed that copy number increase maybe the reason of expression up-regulation. Interestingly, both mRNA and protein expression of GPNMB was higher in cancer tissues than rectal adenoma tissues.

GPNMB is a type I transmembrane protein and overexpressed in several malignant human tissues relative to the corresponding normal tissues. Ectopic overexpression of GPNMB/osteoactivin can promote the metastasis and invasion of glioma, breast and hepatocellular carcinoma [14-17]. EFNA1 was overexpressed in hepatocellular carcinoma and can inhibit growth of malignant mesothelioma by phosphorylating EPHA2 [18,19]. C13orf27 was overexpressed in rectal tumor in our study, but the function of C13orf27 was unknown. PMEPA1 was also identified in our study, which is mapped to 20q13.3 is a TGF-beta inducible gene and encodes a NEDD4 E3 ubiguitin ligase binding protein. PMEPA1 is over-expressed in prostate, breast, renal cell, stomach and rectal carcinomas [20-22]. But little is known about the function of PMEPA1, Further study should be conducted to investigate the roles of the above genes in human colorectal cancer.

Loss of 18q is a common event in colorectal cancer, and 18q deletion and loss of SMAD4 expression are associated with liver metastasis. In colorectal cancer, patients with reduced SMAD4 expression frequently presented an unfavorable survival because of liver metastasis [23-26]. High expression level of SMAD4 reflected significantly longer overall and disease-free survival time than low expression level [27]. Bixiang et al. found that transgenic expression of SMAD4 can significantly reduce the oncogenic potential of MC38 and SW620 cells [28]. Our study confirmed the decreased expression of SMAD4 in rectal cancer.

In summary, we identified EFNA1 (1q), C13orf27 (13q), PMEPA1 (20q), GPNMB (7q) as candidate driving genes of genomic aberrations in rectal cancer. Further study was needed to reveal the mechanisms by which these genes may be involved in the carcinogenesis of the rectum.

## Conclusions

Our data provide detailed information on genomic aberrations present in rectal adenoma or carcinoma, especially both in two groups or only in rectal cancer. Real-time PCR and immunohistochemistry assay selected EFNA1, C13orf27, PMEPA1 and GPNMB as candidate amplification targets. Our results may help to identify the driving genes involved in the adenoma-carcinoma progression.

## Competing interests

The authors declared that they have no competing interest.

## Authors’ contributions

ZYM participated in the collection of specimens. HJJ prepared the genomic DNA and total RNA. SZZ carried out the array CGH study. WBS performed microarray data analysis, LJW, CX and ZY carried out the real-time PCR assay. SL and ZTT performed immunohistochemistry assay. SZZ, WMR, WGQ and ZY participated in the design of the study and performed the statistical analysis. All authors read and approved the final manuscript.

## Pre-publication history

The pre-publication history for this paper can be accessed here:

http://www.biomedcentral.com/1755-8794/5/52/prepub

## Supplementary Material

Additional file 1 **Figure S1.** Comparison of rectal adenoma and carcinoma in number of genomic aberrations. Click here for file
