# Supplementary figures and images for: Genomic profiling of rectal adenoma and carcinoma by array-based comparative genomic hybridization
Source: BMC Med Genomics. 2012 Nov 16;5:52. doi: 10.1186/1755-8794-5-52 (PMC3533962; doi:10.1186/1755-8794-5-52)

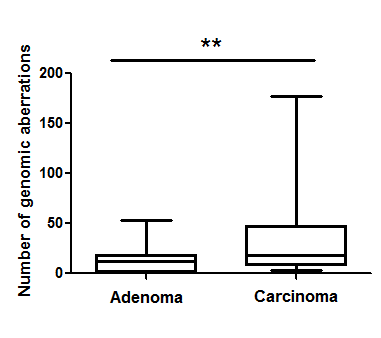

Supplement: Additional file 1 — Figure S1. Comparison of rectal adenoma and carcinoma in number of genomic aberrations. [file 1755-8794-5-52-S1.tiff]
